# Development of a GC-MS/MS method to quantify 120 gut microbiota-derived metabolites

**DOI:** 10.1007/s00216-025-06256-6

**Published:** 2025-12-15

**Authors:** Nikita Denisov, Fabian Springer, Amber Brauer-Nikonow, George Maftei, Georg Zeller, Denise Medeiros Selegato, Michael Zimmermann

**Affiliations:** 1 Molecular Systems Biology Unit, European Molecular Biology Laboratory, Heidelberg, Germany; 2 Center for Infectious Diseases (LUCID), Leiden University, Leiden University Medical Center (LUMC), Leiden, Netherlands

**Keywords:** Gut microbiota, GC-MS/MS, Bacterial metabolites, DNA stabilization buffers

## Abstract

The gut microbiota produces metabolites that are important for host physiology and have critical roles in the development of diseases, such as metabolic disorders, cardiovascular diseases, and cancer. Here, we developed a gas chromatography-coupled tandem mass spectrometry (GC-MS/MS) method for the quantification of 120 volatile and semi-volatile compounds produced by gut bacteria, including short-chain fatty acids, indols, nucleotides, organic acids, and amino acid derivatives. The method is based on multiple-reaction-monitoring (MRM) of each analyte and their respective isotopically labeled internal standard, enabling absolute metabolite quantification between 0.45 pmol and 1 nmol. Applying the method to different tissue samples from germfree and conventionally colonized mice, we illustrate the ability to quantify microbiota-produced metabolites in different sample matrices—plasma, liver, feces, and intestinal content—and at different concentrations. Lastly, we demonstrate that this protocol is capable of quantifying microbiota-derived metabolites in stool samples stored in DNA stabilization buffers that are typically used in sequencing-based microbiome studies. Altogether, the developed GC-MS/MS method adds a valuable analytical tool to quantify microbiota-host metabolic interactions.

**Graphical abstract:**

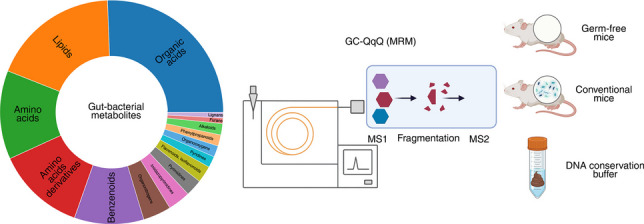

**Supplementary Information:**

The online version contains supplementary material available at 10.1007/s00216-025-06256-6.

## Introduction

The microorganisms residing in the mammalian gastrointestinal tract (gut microbiota) are involved in digestion, general host physiology, and various health states [1]. For example, perturbations of the metabolic interactions between the gut microbiota and their host have been associated with various diseases, including metabolic disorders [2], cardiovascular issues [3, 4], gastrointestinal ailments [5, 6], neurodegenerative conditions [7], and cancer [8–10]. Therefore, metabolomics analyses have become an important tool to study microbiota-host interactions, and modulation of the metabolism of the microbiota has been discussed as treatment and prevention strategies [11, 12]. Gut microbial metabolites are produced through bacterial fermentation of dietary components, through the biosynthesis of novel compounds, and biotransformation of compounds derived from nutrients, the host, or other members of the dense gut microbial community [13]. For example, short-chain fatty acids (SCFAs), such as acetate, propionate, and butyrate, are thought to be key metabolites to understand microbiota-host metabolic interactions [14]. Produced through fiber fermentation under the generally anaerobic conditions in the large intestine, they reach millimolar concentrations [15] in the large intestine and can serve as energy sources for intestinal and hepatic tissues [16]. Other fermentation products, such as lactate and succinate, are involved in microbial cross-feeding and metabolic signaling [17]. Microbial biotransformation products of amino acids, such as the glutamate-derived neurotransmitter gamma-aminobutyric acid (GABA), or tryptophan-derived indole compounds, such as indole-3-propionate and tryptamine, are important signaling molecules between the gut microbiota and the host [18–22].

Numerous protocols have been developed to measure microbiota-produced metabolites in feces, body fluids, and host tissue, with a particular interest in SCFA and other small-molecular-weight metabolites. However, their high volatility causes challenges for the preanalytical sample handling and acquisition in both nuclear magnetic resonance (NMR) spectrometry [23] and liquid chromatography-coupled mass spectrometry approaches [24, 25]. Gas chromatography-based methods are particularly well-suited for the analysis, taking advantage of various protocols to chemically derivatize and stabilize these volatile analytes [26–29].

Quantification of microbiota-produced metabolites has been increasingly incorporated in sequencing-based microbiome studies [30]. Quantification of metabolite concentrations in human and animal models enables direct comparison between studies and allows data translation into mechanistic in vitro models of microbiota-host interactions. In most clinical microbiome studies, the stool samples are typically collected into stabilization buffer to maintain DNA/RNA integrity upon storage and shipment. However, the various detergents, high salt concentrations, and other (often unknown) buffer additives are often incompatible with liquid chromatography systems, hampering accurate metabolic measurements.

Here, we developed a workflow to quantify 120 gut microbiota-produced metabolites, many of which have been reported to play a role in metabolic host-microbiota interactions. To this aim, we established multiple-reaction-monitoring (MRM) assays on a gas chromatography-coupled tandem mass spectrometer (GC-MS/MS) for each of the 120 metabolites and 52 matching isotopically labeled internal standards. We then applied the developed method on intestinal, plasma, and liver samples of germ-free and conventional mice to quantify the contribution of the microbiota to intestinal and systemic metabolite levels. Additionally, we demonstrate that the developed method is compatible with DNA/RNA conservation buffers and that the majority of metabolites can be quantified in intestinal samples stored in such buffers.

## Experimental section

### Chemicals and preparation of metabolite standards

LC-MS-grade (ChemSolute®) pyridine, ethanol, methanol, and water were purchased from TH.GEYER (Renningen, Germany). N-tert-butyldimethylsilyl-N-methyltrifluoroacetamide (CAS:77,377−52−7) and methoxyamine HCl (CAS:61-16−5) were purchased from Sigma-Aldrich (St. Louis, MO) (Supplementary Table S1). Helium was used as carrier gas for GC-MS/MS (Helium 5.0, Messer SE & Co. KGaA, Germany). Argon was used as collision gas for GC-MS/MS (Argon 5.0, Messer SE & Co. KGaA, Germany). A 0.1M NaOH solution was prepared by diluting 10M NaOH in water. Invitek stabilization buffer (Item No.: 1038111200) and OMNIgene gut stabilization buffer (Ottawa, ON) were purchased from Invitek Diagnostics and DNA Genotek, respectively. Metabolite standards were purchased at the highest purity available, dissolved in ethanol, water, or methanol, based on metabolite solubilities at a concentration of 10 mM (Supplementary Table S2). These chemical standards were combined into two mixtures at a concentration of 500 µM and 166.6 µM so that none of the pools contains analytes with the same nominal mass. Before metabolite extraction, an internal standard mixture (IS mix) of isotopically labeled organic acids (250 µM), amino acids (500 µM), hexanoic acid, valeric acid, isovaleric acid, caproic acid, and indole-propionic acid (10 mM) was spiked into each sample (Supplementary Table S3).

### Metabolite extraction from animal samples

The liver, feces, and intestinal content were defrosted, and the weights were recorded and adjusted to 50–200 mg. Each tissue was added to a 2 mL O-ring tube (HS10060, Heathrow Scientific HEA10060), together with 200 µL of 0.1 mm zirconia/silica beads and 500 µl of a solvent mixture (H₂O:ACN:MeOH, 25:50:50, v/v/v). Samples were homogenized by bead-beating for 5 min at 2400 rpm (Mini-BeadBeater 96, product No.:1001EUR, BioSpec Products, USA). The lysed samples were kept in the freezer for 1 h and then centrifuged at 10,000g at 4 °C for 12 min. Forty microliters of supernatant was transferred to a 96-well plate and derivatized as described below. Animal derived plasma and NIST 1950 serum (100 µl) was deproteinised with 200 µl (MeOH:H_2_0, 9:1, v:v) (1:3,  −20 °C), and 20 µl of supernatant was derivatized as described below.

### Metabolite derivatization

Twenty microliters of each sample was added to a mixture of 12.8 µL IS mix and 25 µL of 0.1M sodium hydroxide. Samples were dried in a speed-vac (Genevac EZ-2 4.0 Series Centrifugal Evaporators, © Avantor, Inc.) at 30 °C. A fresh solution of methoxyamine HCl in pyridine (MeOX) was prepared by dissolving 20 mg of methoxyamine HCl in 1 mL of pyridine under vortexing. Twenty microliters of MeOX was added to dried samples, resuspended, and covered with aluminum foil. Samples were incubated at 60 °C for 60 min. Twenty microliters of N-tert-butyldimethylsilyl-N-methyltrifluoroacetamide was then added to the samples, samples were resuspended, and the plates were covered with aluminum foil. Samples were incubated at 60 °C for 60 min and centrifuged for 5 min at 16,000 rpm, and 40 µL of supernatant was transferred to glass vials with inlets (screw top, with fixed insert, amber, 300 µL insert volume. Vial size: 12 × 32 mm, Part No.: 5188-6592, Agilent).

### GC-MS/MS settings

GC-MS/MS acquisitions were performed on a Shimadzu instrument (TQ8040) using a Zebron ZB-5ms (30 m × 0.25 mm × 0.25 µm) column applying the following settings: injection in split mode 1:10, injection temperature 250 °C, flow control mode–linear velocity 40.1 cm/s, pressure 83.3 kPa, total flow 17.9 mL/min, purge flow 5.0 mL/min, column flow 1.17 mL/min, and column oven starting temperature 90 °C (for 1.5 min), followed by a temperature gradient from 90 to 320 °C at a rate of 15 °C/min and kept at 320 °C until 25-min total run time. Injection volume was set to 1 μL. Ion source temperature was kept at 230 °C, interface temperature at 280 °C, and solvent cut time at 0 min. Full-scan mode was acquired from 50 to 500 m/z, EI at 70 eV. MRM settings were applied as described below.

### Sensitivity and quantification

Dilution series were prepared using two separate mixtures of chemical standards: one composed of 30 organic acids (Cambridge Isotope Laboratories, MSK-OA) and the second composed of the remaining 90 compounds, weighted and mixed manually. Dilution series started at a concentration of 12.5 µM (12.5 pmol injected) and 25 µM (25 pmol injected) for the first and second mixture, respectively. 1:3 dilution steps were performed ten times (final concentration range of 1000 µM and 0.01 µM). Fifty-two isotopically labeled compounds were used as internal standards to enable absolute quantification of the analytes (Supplementary Table S3). A total of 12.8 µL of 25 µM internal standards mixture was added to each sample to reach a final concentration of 8 µM in the derivatized solution. For each analytical batch, four blank extractions (containing no biological sample) were processed alongside study samples. Blanks underwent the full homogenization, extraction, and derivatization workflow and were measured throughout the measurement batch. The signals of blank samples were inspected to assess baseline noise and the absence of contamination or carryover. No blank subtraction was performed; blank measurements were used solely for quality control assessment. Signals in blank samples prepared for concentration to signal calibration curves were used to determine LOD and LOQ of each metabolite (in particular of SCFAs).

### Precision assessment

To evaluate intraday and interday precision, cecum and plasma samples were collected from 15 CONV and 8 GF animals of mixed sex and pooled within animal group. On three subsequent days, four independent aliquots of the pooled samples were extracted and derivatized as a batch.

- Day 1: Batch 1 was prepared, and each of the four replicates was injected twice to assess intraday variability.
- Day 2: Batch 2 was freshly extracted and derivatized, and each replicate was injected twice; in addition, Batch 1 was injected a third time for the interday measurement variability assessment.
- Day 3: Batch 3 was prepared and injected twice, and Batch 2 was injected a third time.
- Day 4: Batch 3 was injected a third time to complete the interday measurements. This design enabled evaluation of intraday variability (duplicate injections of freshly prepared replicates) and interday variability (third injections of batches on subsequent days).

### Animal experiments

Germ-free (GF) B6NTac mice were obtained from Taconic Biosciences and maintained and bred in gnotobiotic isolators (CbC) with a 12-h light/dark cycle. GF status was monitored by PCR and culture-based methods. Specific pathogen-free (conventional mice) C57BL/6J were originally obtained from The Jackson Laboratory, and mice were routinely tested and negative for *Helicobacter *spp. and other known murine pathogens. Mice were provided with autoclaved chow (1318 P FORTI, Altromin) ad libitum. Animals of mixed sex between 13 and 20 weeks of age were used for all experiments (Supplementary Table 4). GF and conventional animals were singly housed for 14 days before they were euthanized by CO_2_. Plasma, liver, and fecal samples, as well as cecal, duodenal, jejunal, ileal, and colon contents were collected, snap-frozen, and stored at −70 °C until further processing. Mouse experiments were approved by IACUC (license number 21-002_HD_MZ).

### Data analysis

Chromatographic data was acquired and processed using Shimadzu LabSolutions GC-MS software (version 5.131, Shimadzu Corporation, Kyoto, Japan). Peak detection and integration were performed using the software default settings with manual adjustment when necessary to ensure consistency across samples. After integration, data were exported as tab-delimited text (.txt) files for further statistical analysis in R (Ver. 4.2.2). Statistical data analyses were performed in R (R version 4.2.2 (2022-10−31)). All code is available on GitHub (https://github.com/ZimmermannLab/gcms-gut-metab-quant).

## Results and discussion

**Fig. 1:**
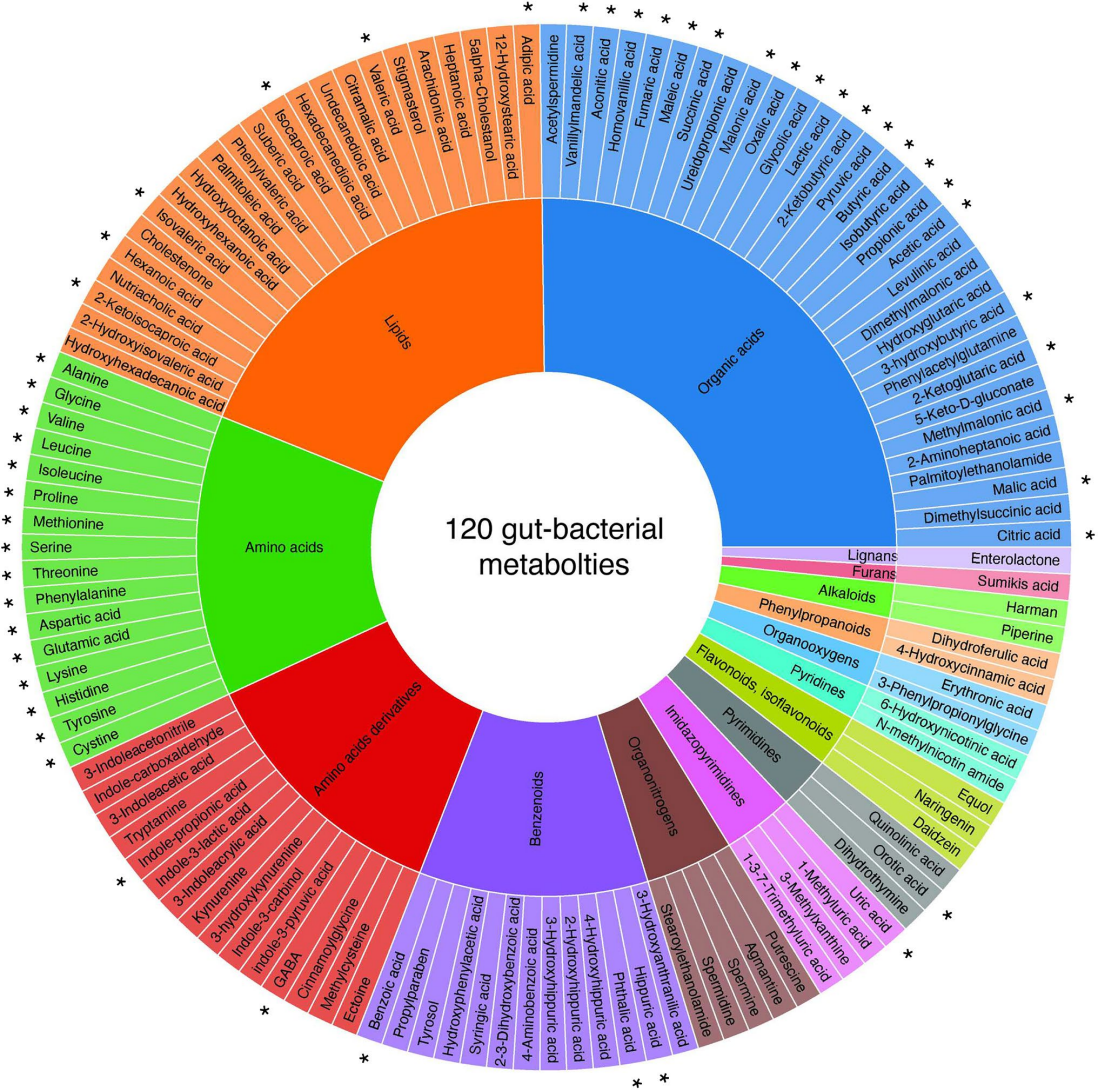
Metabolites included in the method. Asterisks (*) indicate metabolites with matched isotopically labeled internal standards used for absolute quantification. Colors depict chemical classes defined by MiMeDB [31]

### Gut microbial metabolite library preparation and metabolite derivatization

To establish a targeted metabolomics method, we assembled a library of 120 chemical standards of metabolites that are produced by the human gut microbiota and that span multiple metabolite classes as defined by the Microbial Metabolites Database (MiMeDB) [31] (Fig. 1, Table 1, and Supplementary Table S2). The library includes the short-chain fatty acids (SCFAs) acetate, propionate, and butyrate, which are produced during gut bacterial fiber fermentation, serve as important energy substrates for colonocytes and hepatocytes, and can function as modulators of immune regulation [32, 33]. We also included branched-chain fatty acids (BCFAs) [34]; organic acids, such as lactate [35] and succinate [36]; and indoles [37] derived from tryptophan metabolism, all of which are involved in microbial cross-feeding and signaling [38]. Additionally, amino acids [39] and some of their derivatives, including GABA [40], a neurotransmitter linked to gut-brain communication, were part of the library (Fig. 1, Table 1). The selected metabolites range from 60.05 Da (acetic acid) to 412.69 Da (stigmasterol). Further, these compounds have a broad range of boiling points—from low (e.g., short-chain fatty acids) to high (e.g., non-volatile amino acids and dicarboxylic acids, such as succinate and fumarate). In addition, we included isotopically labeled compounds for 52 of the metabolites as internal standards (IS), enabling metabolite quantification (Fig. 1, Table 1, Supplementary Table S3).

To facilitate gas chromatographic separation, we chemically derivatized the metabolites as previously reported by Gu et al. [41]. In brief, we first basified the samples with NaOH to decrease the volatility of organic acids and, hence, enable sample drying under vacuum. Prior to GC-MS/MS analysis, we then performed methoxymation and silylation (using methoxyamine and N-tert-butyldimethylsilyl-N-methyltrifluoroacetamide—MTBSTFA), respectively, to enhance volatility and thermal stability. A key advantage of using MTBSTFA is that the derivatization reaction can be performed at 60 °C, without the requirement of ultrasonication [42]. Moreover, the chemical replacement of polar and reactive functional groups with less polar and more thermally stable groups allows direct injection into the GC-MS instrument without the need of any further extraction steps [43].

### Optimization of MRM assays

**Fig. 2:**
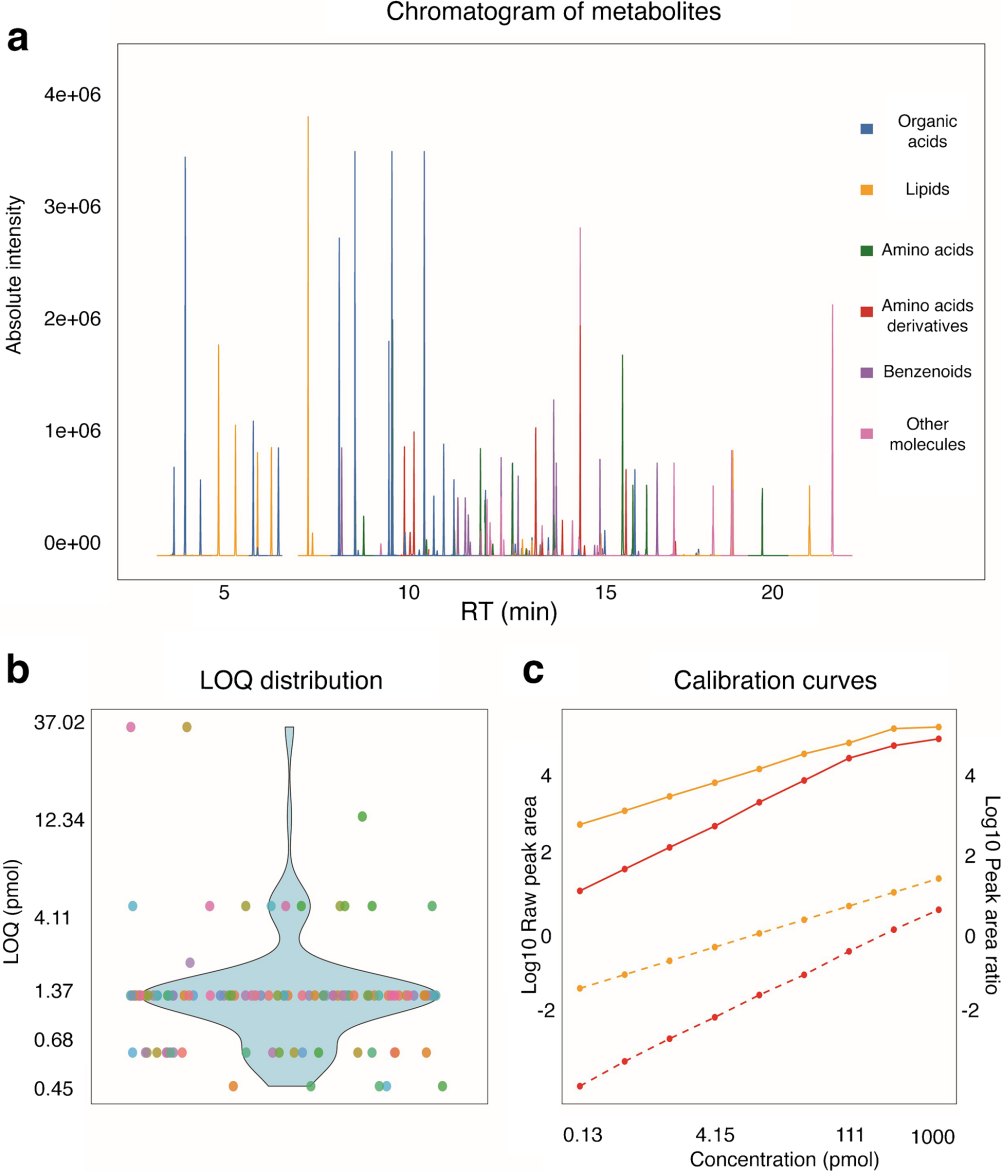
**a** Chromatogram and **b** limit of quantification of 120 gut bacteria-derived metabolites. Colors indicate the MiMeDB metabolite class. **c** Calibration curves of valeric acid (orange, solid line) corrected with ^2^H₉-valeric acid as internal standard (orange, dashed line) and of tryptamine without internal standard correction (red, solid line) and with d2-indole-propionic acid for internal standard correction (red, dashed line)

We optimized the temperature gradient of the gas chromatography separation for the separation of all metabolites. To this end, all 120 metabolites were injected separately and as a mixture to determine their retention times, which ranged from 2.35 to 24.90 min (Fig. 2a and Table 1) in the final 25-min run.

To optimize the MRM parameters of the triple quadrupole (QqQ) instrument, we then selected one or two precursor ions for each compound and isotopically labelled internal standards. To this aim, we injected each metabolite eight times and ramped the collision energy (CE) of Q2 from 3 to 45 eV to determine specific MS/MS-fragments and the CE resulting in their highest occurrence. Using the Smart Database (GCMSsolution Ver. 4.22), the optimized MRM parameters were managed and together with their respective retention time assembled to the final GC-MS/MS method (Table 1). Confirmation ions were selected to help quantification through improved identification of the peaks upon splitting or retention time shifts.

**Table 1:** Key parameters for TBDMS-derivatized metabolites and their corresponding isotopically labeled internal standards, including retention time, MRM transitions, collision energy (CE), limit of quantification (LOQ), range of linearity of quantification, and limit of detection (LOD). *IS, internal standard used for the quantification of a specific metabolite.

№	Name	RT, mins	MRM transition (target ion)	CE	MRM transition (confirmation ion)	CE	LOQ, pmol	Linear range, pmol	LOD, pmol	IS*
1	Acetic acid	2.35	117.00 > 75.00	9	159.00 > 117.00	9	12,34	12,34–1000	1,37	IS 1
IS 1	Acetic acid, ^13^C₂	2.35	119.00 > 75.00	9		9				
2	Propionic acid	3.18	131.00 > 75.00	9			12,34	12,34–1000	1,37	IS 2
IS 2	Propionic acid, ^13^C₃	3.18	134.00 > 75.00	9		9				
3	Isobutyric acid	3.38	145.00 > 75.10	9			4,11	4,11–1000	1,37	IS 3
IS 3	Isobutyric acid, ^13^C₄	3.38	149.00 > 75.10	9						
4	Butyric acid	3.74	145.00 > 75.10	15			1,37	4,11–1000	1,37	IS 4
IS 4	Butyric acid, ^13^C₄	3.74	149.00 > 75.10	15						
5	Isovaleric acid	4.16	159.00 > 75.00	15			1,37	1,37–1000	0,45	IS 5
IS 5	Isovaleric acid, ^2^H₉	4.16	168.00 > 75.00	15						
6	Valeric acid	4.57	159.00 > 75.00	9			1,37	1,37–1000	0,45	IS 6
IS 6	Valeric acid, ^2^H₉	4.57	168.00 > 75.00	9						
7	Pyruvic acid	5.09	174.00 > 74.00	21			0,68	0,68–500	0,22	IS 7
IS 7	Pyruvic acid, ^13^C₃	5.09	177.00 > 74.00	21						
8	Isocaproic acid	5.15	173.00 > 75.00	24			0,45	0,45–1000	0,15	IS 8
IS 8	Isocaproic acid, ^2^H₁₁	5.15	184.00 > 75.00	24						
9	Hexanoic acid	5.51	173.00 > 75.00	12			0,45	0,45–1000	0,15	IS 9
IS 9	Hexanoic acid, ^2^H₃	5.51	176.00 > 75.00	12						
10	2-Ketobutyric acid	5.55	188.00 > 74.00	21			0,68	0,68–500	0,22	IS 10
IS 10	2-Ketobutyric acid, ^13^C₄	5.55	192.00 > 74.00	21						
11	Keto-isovaleric acid	5.81	202.00 > 89.00	9			0,68	0,68–500	0,22	IS 11
IS 11	Keto-isovaleric acid, ^13^C₅	5.81	207.00 > 89.00	9						
12	Heptanoic acid	6.44	187.00 > 75.00	24			0,45	0,45–1000	0,15	IS 9
13	2-Ketoisocaproic acid	6.56	216.00 > 89.00	15	105.00 > 77.00	15	1,37	1,37–1000	0,45	IS 12
IS 12	2-Ketoisocaproic acid, ^13^C₆	6.56	222.00 > 89.00	15						
14	Lactic acid	7.20	261.00 > 147.00	15			1,37	1,37–1000	0,45	IS 13
IS 13	Lactic acid, ^13^C₃	7.20	264.00 > 147.00	15						
15	Glycolic acid	7.50	247.00 > 73.00	24			1,37	1,37–1000	0,45	IS 14
IS 14	Glycolic acid, ^13^C₂	7.50	249.00 > 73.00	24						
16	Benzoic acid	7.60	179.00 > 105.00	15			0,68	0,68–500	0,22	IS 15
IS 15	Benzoic acid, ^13^C_6_	7.60	185.00 > 111.00	15						
17	L-Alanine	7.61	232.00 > 147.00	15	260.00 > 147.00	24	1,37	1,37–1000	0,45	IS 16
IS 16	L-Alanine, ^13^C₃^15^N	7.61	235.00 > 147.00	15						
18	Oxalic acid	7.70	261.00 > 73.00	21			1,37	1,37–1000	0,45	IS 17
IS 17	Oxalic acid, ^13^C₂	7.70	263.00 > 73.00	21	191.00 > 147.00	15				
19	Glycine	7.98	218.00 > 147.00	15	119.00 > 78.00	15	1,37	1,37–1000	0,45	IS 18
IS 18	Glycine, ^13^C₂^15^N	7.98	220.00 > 147.00	15						
20	3-Hydroxybutyric acid	8.20	275.00 > 73.00	24			0,68	0,68–500	0,22	IS 19
IS 19	3-Hydroxybutyric acid, ^13^C_4_	8.20	279.00 > 73.00	24	231.00 > 147.00	15				
21	Malonic acid	8.60	275.00 > 73.00	33	189.00 > 147.00	15	0,68	0,68–500	0,22	IS 20
IS 20	Malonic acid, ^13^C₃	8.60	278.00 > 73.00	33						
22	N-methylnicotinamide	8.60	193.00 > 119.00	21			1,37	1,37–1000	0,45	IS 21
23	L-Valine	8.67	260.00 > 147.00	15	288.00 > 147.00	21	1,37	1,37–1000	0,45	IS 21
IS 21	L-Valine, 13C	8.67	265.00 > 147.00	15						
24	Methylmalonic acid	8.74	289.00 > 147.00	15			0,68	0,68–500	0,22	IS 22
IS 22	Methylmalonic acid, ^13^C_4_	8.74	293.00 > 147.00	15	199.00 > 130.00	15				
25	Dimethylmalonic acid	8.78	303.00 > 147.00	15	189.00 > 147.00	15	1,37	1,37–1000	0,45	IS 20
26	L-Leucine	8.95	274.00 > 147.00	15			1,37	1,37–1000	0,45	IS 23
IS 23	L-Leucine, ^13^C₆^15^N	9.01	280.00 > 147.00	15						
27	Isoleucine	9.21	274.00 > 147.00	15	291.00 > 188.00	27	1,37	1,37–1000	0,45	IS 24
IS 24	Isoleucine, ^13^C₆^15^N	9.23	280.00 > 147.00	15	287.00 > 188.00	27				
28	Ectoine	9.39	97.00 > 68.00	15			12,34	12,34–1000	4,11	IS 43
29	Dimethylsuccinic	9.49	289.00 > 73.00	21			1,37	1,37–1000	0,45	IS 28
30	L-Proline	9.49	258.00 > 147.00	15			1,37	1,37–1000		IS 25
IS 25	L-Proline, ^13^C₅^15^N	9.49	263.00 > 147.00	15						
31	Maleic acid	9.50	287.00 > 147.00	15			2,05	2,05–500	0,68	IS 26
IS 26	Maleic acid, ^13^C₄	9.50	291.00 > 147.00	15						
32	GABA	9.51	274.00 > 147.00	24	291.00 > 147.00	15	0,45	0,45–1000	0,15	IS 27
IS 27	GABA, ^2^H₆	9.51	280.00 > 147.00	24						
33	Succinic acid	9.56	289.00 > 73.00	24			1,37	1,37–1000	0,45	IS 28
IS 28	Succinic acid, ^13^C_4_	9.56	293.00 > 73.00	24						
34	Putrescine	9.76	145.00 > 74.00	15	128.00 > 59.00	15	0,45	0,45–1000	0,15	IS 27
35	Fumaric acid	9.80	287.00 > 73.00	24			0,68	0,68–500	0,22	IS 29
IS 29	Fumaric acid, ^13^C₄	9.80	291.00 > 73.00	24	235.00 > 117.00	15				
36	2-Aminoheptanoic acid	10.07	214.00 > 73.00	15	228.00 > 73.00	21	1,37	1,37–1000	0,45	IS 29
37	2-Hydroxyoctanoic acid	10.37	331.00 > 147.00	15	237.00 > 75.00	27	1,37	1,37–1000	0,45	IS 29
38	5-Hydroxyhexanoic acid	10.37	303.00 > 147.00	15	323.00 > 73.00	39	1,37	1,37–1000	0,45	IS 29
39	Methylcysteine	10.41	278.00 > 147.00	21	317.00 > 73.00	39	1,37	1,37–1000	0,45	IS 25
40	Phenylvaleric acid	10.57	235.00 > 75.00	15			1,37	1,37–1000	0,45	IS 6
41	Indole-6-carboxaldehyde	10.58	383.00 > 73.00	21			1,37	1,37–1000	0,45	IS 43
42	Indole-3-acetonitrile	10.70	383.00 > 73.00	27	194.00 > 92.00	27	1,37	1,37–1000	0,45	IS 43
43	Propylparaben	10.80	237.00 > 151.00	15			4,11	4,11–1000	1,37	IS 43
44	Adipic acid	10.81	111.00 > 83.00	6	156.00 > 75.00	15	0,68	0,68–500	0,22	IS 30
IS 30	Adipic acid, ^13^C_6_	10.81	117.00 > 83.00	6						
45	Levulinic acid	10.90	315.00 > 81.00	21			1,37	1,37–1000	0,45	IS 30
46	L-Methionine	10.91	292.00 > 147.00	18			1,37	1,37–1000	0,45	IS 31
IS 31	L-Methionine, ^13^C₅^15^N	10.91	297.00 > 147.00	18						
47	4-Aminobenzoic acid	10.96	194.00 > 120.00	15			1,37	1,37–1000	0,45	IS 43
48	L-Serine	11.03	362.00 > 147.00	18			1,37	1,37–1000	0,45	IS 32
IS 32	L-Serine, ^13^C₃^15^N	11.03	365.00 > 147.00	18	313.00 > 73.00	33				
49	Alpha-ketoglutarate	11.19	346.00 > 73.00	24	219.00 > 177.00	15	0,68	0,68–500	0,22	IS 33
IS 33	Alpha-ketoglutarate, ^13^C_4_	11.19	350.00 > 73.00	24	154.00 > 127.00	15				
50	Agmatine	11.20	284.00 > 184.00	24			0,45	0,45–1000	0,15	IS 34
51	L-Threonine	11.21	303.00 > 73.00	15			1,37	1,37–1000	0,45	IS 34
IS 34	L-Threonine, ^13^C₄^15^N	11.21	307.00 > 73.00	15	266.00 > 73.00	21				
52	5-Keto-D-gluconate	11.23	156.00 > 75.00	21			1,37	1,37–1000	0,45	IS 33
53	Sumikis acid	11.27	313.00 > 147.00	15	423.00 > 147.00	15	1,37	1,37–1000	0,45	IS 43
54	Tyrosol	11.43	309.00 > 73.00	27	419.00 > 403.00	15	1,37	1,37–1000	0,45	IS 43
55	Harman	11.45	182.00 > 154.00	27			4,11	4,11–1000	1,37	IS 43
56	6-Hydroxynicotinic acid	11.54	310.00 > 73.00	27	323.00 > 73.00	39	1,37	1,37–1000	0,45	IS 43
57	L-Phenylalanine	11.70	308.00 > 147.00	15	341.00 > 236.00	39	1,37	1,37–1000	0,45	IS 35
IS 35	L-Phenylalanine, ^13^C₉^15^N	11.70	317.00 > 147.00	15	356.00 > 73.00	24				
58	Malic acid	11.70	287.00 > 73.00	27	303.00 > 129.00	15	0,68	0,68–500	0,22	IS 36
IS 36	Malic acid, ^13^C₄	11.70	291.00 > 73.00	27						
59	Acetylspermidine	11.80	126.00 > 98.00	9	98.00 > 83.00	15	4,11	4,11–1000	1,37	IS 35
60	Citramalic acid	11.84	301.00 > 73.00	21			1,37	1,37–1000	0,45	IS 36
61	Hydroxyphenylacetic acid	11.88	323.00 > 75.00	27			1,37	1,37–1000	0,45	IS 37
62	Phthalic acid	11.96	337.00 > 73.00	24	288.00 > 89.00	15	0,68	0,68–500	0,22	IS 37
IS 37	Phthalic acid, ^13^C_4_	11.96	341.00 > 73.00	24						
63	Ureidopropionic acid	11.98	129.00 > 75.00	15	169.00 > 75.00	15	1,37	1,37–1000	0,45	IS 43
64	Hippuric acid	12.01	105.00 > 77.00	15			0,68	0,68–500	0,22	IS 38
IS 38	Hippuric acid, ^13^C_6_	12.01	111.00 > 83.00	15						
65	L-Aspartic acid	12.05	390.00 > 73.00	15			1,37	1,37–1000	0,45	IS 39
IS 39	L-Aspartic acid, ^13^C₄^15^N	12.05	394.00 > 73.00	15						
66	Quinolinic acid	12.14	338.00 > 73.00	27			1,37	1,37–1000	0,45	IS 43
67	Suberic acid	12.19	345.00 > 73.00	27			1,37	1,37–1000	0,45	IS 40
68	3-Indoleacetic acid	12.34	232.00 > 130.00	24	324.00 > 73.00	27	1,37	1,37–1000	0,45	IS 43
69	Hydroxyglutaric acid	12.37	433.00 > 147.00	24			0,68	0,68–500	0,22	IS 40
IS 40	Hydroxyglutaric acid, ^13^C₅	12.37	438.00 > 147.00	15						
70	Tryptamine	12.38	144.00 > 73.00	15	261.00 > 73.00	27	1,37	1,37–1000	0,45	IS 43
71	Homovanillic acid	12.60	281.00 > 75.00	24			0,68	0,68–500	0,22	IS 41
IS 41	Homovanillic acid, ^13^C_2_	12.60	283.00 > 75.00	24	105.00 > 77.00	21				
72	3-Hydroxyanthranilic acid	12.69	192.00 > 136.00	27			0,45	0,45–1000	0,15	IS 43
73	L-Glutamic acid	12.70	272.00 > 147.00	18	375.00 > 73.00	39	1,37	1,37–1000	0,45	IS 42
IS 42	L-Glutamic acid, ^13^C₅^15^N_2_	12.70	277.00 > 147.00	18	324.00 > 267.00	9				
74	Hydroxycinnamic acid	12.76	335.00 > 73.00	27			1,37	1,37–1000	0,45	IS 43
75	Spermidine	12.78	474.00 > 73.00	27			1,37	1,37–1000	0,45	IS 43
76	Phenylpropionic acid	12.91	264.00 > 105.00	15			1,37	1,37–1000	0,45	IS 43
77	Citrullin	13.03	286.00 > 147.10	15	258.00 > 147.10	15	1,37	1,37–1000	0,45	IS 43
78	Indole-propionic acid	13.05	246.00 > 117.00	9			1,37	1,37–1000	0,45	IS 43
IS 43	Indole-propionic acid, ^2^H₂	13.05	248.00 > 117.00	12						
79	Palmitoleic acid	13.06	311.00 > 75.10	27			1,37	1,37–1000	0,45	IS 43
80	Erythronic acid	13.10	301.00 > 73.00	27			4,11	4,11–1000	1,37	IS 45
81	1,3,7-Trimethyluric acid	13.28	267.00 > 1000.00	27	369.00 > 223.00	27	1,37	1,37–1000	0,45	IS 51
82	L-Lysine	13.29	300.00 > 147.00	18			1,37	1,37–1000	0,45	IS 44
IS 44	L-Lysine, ^13^C₆^15^N₂	13.29	307.00 > 147.00	18	367.00 > 179.00	21				
83	Aconitic acid	13.31	459.00 > 73.00	24	223.00 > 75.00	27	0,68	0,68–500	0,22	IS 45
IS 45	Aconitic acid, ^13^C_3_	13.31	462.00 > 73.00	24	378.00 > 147.00	27				
84	Syringic acid	13.35	369.00 > 297.00	15	388.00 > 73.00	21	1,37	1,37–1000	0,45	IS 46
85	Orotic acid	13.35	441.00 > 73.00	24	461.00 > 147.00	15	0,68	0,68–500	0,22	IS 46
IS 46	Orotic acid, ^15^N₂	13.35	443.00 > 73.00	24	439.00 > 73.00	39				
86	Dihidroferulic acid	13.38	179.00 > 149.00	27			1,37	1,37–1000	0,45	IS 46
87	Tricarballylic acid	13.50	461.00 > 94.00	21			1,37	1,37–1000	0,45	IS 47
88	Cinnamoyglycine	13.51	206.00 > 75.00	15			1,37	1,37–1000	0,45	IS 44
89	Dihydroxybenzoic acid	13.82	439.00 > 365.00	21	594.00 > 497.00	42	1,37	1,37–1000	0,45	IS 37
90	Undecanedioic acid	13.85	387.00 > 73.00	21	591.00 > 497.00	42	1,37	1,37–1000	0,45	IS 47
91	Vanillylmandelic acid	13.96	381.00 > 73.00	24	417.00 > 73.00	21	0,68	0,68–500	0,22	IS 47
IS 47	Vanillylmandelic acid, ^13^C_6_	13.96	387.00 > 73.00	24	192.00 > 174.00	15				
92	3-Methyluric acid	14.30	300.00 > 147.00	21			1,37	1,37–1000	0,45	IS 43
93	L-Histidine	14.40	196.00 > 73.00	24	361.00 > 75.00	33	1,37	1,37–1000	0,45	IS 48
IS 48	L-Histidine, ^13^C₆^15^N_3_	14.40	202.00 > 73.00	24						
94	Citric acid	14.49	459.00 > 147.00	24			0,68	0,68–500	0,22	IS 49
IS 49	Citric acid, ^13^C_3_	14.49	462.00 > 147.00	24						
95	Kynurenine	14.51	120.00 > 92.00	9	443.00 > 147.00	27	1,37	1,37–1000	0,45	IS 43
96	Indole-3-carbinol	14.54	244.00 > 73.00	15	235.00 > 73.00	27	1,37	1,37–1000	0,45	IS 43
97	Arachidonic acid	14.59	129.00 > 75.00	9			4,11	4,11–1000	1,37	IS 51
98	Indole-3-lactic acid	14.63	193.00 > 73.00	21			1,37	1,37–1000	0,45	IS 43
99	L-Tyrosine	14.63	302.00 > 73.00	18	172.00 > 55.00	21	1,37	1,37–1000	0,45	IS 50
IS 50	L-Tyrosine, ^13^C₉^15^N	14.63	305.00 > 73.00	18	339.00 > 131.00	15				
100	3-Hydroxypalmitic acid	14.75	327.00 > 73.00	27	567.00 > 171.00	15	1,37	1,37–1000	0,45	IS 30
101	2-Hydroxyhippuric acid	14.76	569.00 > 214.00	33			1,37	1,37–1000	0,45	IS 30
102	4-Hydroxyhippuric acid	15.30	235.00 > 73.00	21	455.00 > 149.00	21	1,37	1,37–1000	0,45	IS 30
103	Palmitoyl ethanolamide	15.54	356.00 > 118.00	15	129.00 > 75.00	15	1,37	1,37–1000	0,45	IS 43
104	Uric acid	15.67	567.00 > 73.00	15	384.00 > 57.00	21	1,37	1,37–1000	0,45	IS 51
IS 51	Uric acid, ^15^N₂	15.67	569.00 > 73.00	15	115.00 > 89.00	21				
105	3-Indoleacrylic acid	15.75	358.00 > 73.00	15			1,37	1,37–1000	0,45	IS 43
106	Phenylacetil-glutaric acid	15.86	592.00 > 427.00	15			1,37	1,37–1000	0,45	IS 43
107	2-Hydroxystearic acid	15.93	471.00 > 73.00	27	413.00 > 119.00	39	1,37	1,37–1000	0,45	IS 30
108	Hexadecanedioic acid	16.32	457.00 > 73.00	27	217.00 > 186.00	39	1,37	1,37–1000	0,45	IS 30
109	Stearoylethanolamide	16.49	384.00 > 118.00	15	445.00 > 161.00	15	1,37	1,37–1000	0,45	IS 43
110	Piperine	16.50	201.00 > 115.00	21	482.00 > 425.00	21	4,11	4,11–1000	1,37	IS 43
111	Indole-pyruvic acid	16.90	488.00 > 147.00	33	432.00 > 73.00	27	4,11	4,11–1000	1,37	IS 43
112	L-Cystine	17.10	302.00 > 73.00	15	469.00 > 83.00	21	0,68	0,68–500	0,22	IS 52
IS 52	L-Cystine, ^13^C₆^15^N₂	17.10	305.00 > 73.00	15	561.00 > 159.00	21				
113	3-Hydroxy-DL-Kynurenine	17.11	322.00 > 73.00	27	250.00 > 73.00	27	1,37	1,37–1000	0,45	IS 43
114	Spermine	17.74	531.00 > 112.00	21			1,37	1,37–1000	0,45	IS 43
115	Cholestenone	17.80	413.00 > 137.00	27			1,37	4,11–1000	0,45	IS 51
116	Enterolactone	18.14	469.00 > 164.00	39			4,11	4,11–1000	1,37	IS 51
117	Cholestan-3-ol	18.82	445.00 > 75.00	27			1,37	1,37–1000	0,45	IS 30
118	Daidzen	19.41	425.00 > 397.00	21			4,11	4,11–1000	1,37	IS 43
119	Stigmasterol	19.69	83.00 > 55.00	3			1,37	1,37–1000	0,45	IS 51
120	Nutriacholic acid	24.72	159.00 > 129.00	21			1,37	1,37–1000	0,45	IS 51

### Quantification of metabolites

The limit of detection (LOD) was defined as the lowest concentration at which the signal for a given metabolite could be reliably distinguished from background noise, corresponding to a signal-to-noise ratio of greater than three. We found an LOD of 0.05 pmol for organic acids, lipids, and lipid-like molecules; 12.34 pmol for short-chain fatty acids (SCFAs); 0.15 pmol for amino acids and their derivatives; and 4.11 pmol for flavonoids, alkaloids, furanoid ligands, and organooxygen compounds (Table 1). For the limit of quantitation (LOQ), we report the concentration for which the signal to noise ratio was greater than 10 (Fig. 2b).

For the absolute quantification of metabolites, we calculated the ratio between the peak area of metabolites and their corresponding isotopically labeled internal standards and compared the value to the corresponding calibration curves. In the absence of a matching internal standard compound, we selected a structurally similar internal standard with close retention time to the analyte (Table 1) to determine metabolite concentrations. This strategy extended the linear range of quantification, as demonstrated for valeric acid and tryptamine, whose calibration curves were corrected using ^2^H₉-valeric acid and ^2^H-indole-propionic acid, respectively (Fig. 2c). Following this approach, we achieved a linear quantification range of 0.68 to 500 pmol for organic acids and 1.37 to 1000 pmol for amino acids, branched-chain fatty acids, indole derivatives, and lipid-like molecules. Some compounds, such as short-chain fatty acids (4.11–1000 pmol), ectoine (12.34–1000 µM), propylparaben, piperine, arachidonic acid, erythronic acid, and enterolactone (0.103–1000 pmol), exhibited narrower linear ranges.

To validate the method, we analyzed the certified reference plasma material NIST SRM 1950. Four 100 µL aliquots of the NIST plasma were extracted and derivatized as independent replicates following the developed protocol for sample extraction, derivatization, and GC-MS/MS measurements. Accuracy was evaluated for metabolites for which reference concentrations are available: 15 metabolites with certified values reported by NIST and an additional 9 metabolites with literature values reported by Mandal et al. [44]. These quantified metabolites span four orders of magnitude in concentration (from 0.3 to 2600 µM), allowing the assessment of method accuracy across a broad concentration range in a physiological matrix. For both the NIST-certified set and the literature-reported set of metabolites, the concentrations measured with our method were within the respective reference ranges (Table 2, Supplementary Table S14). These results demonstrate robust analytical accuracy. Further, these analyses suggest using NIST SRM 1950 as external quality-control material for future measurement batches to ensure inter-batch and inter-study comparability.

**Table 2:** Quantified metabolites in NIST SRM 1950 plasma and comparison to reference concentrations and concentrations recently reported [44]. Standard deviations are based on four independent replicate measurements

Metabolites	Concentration ± SD (μM) **Measured**	Concentration ± SD (μM) **Reference **[44]	Concentration ± SD (μM) **NIST**
Acetic acid	99.56 ± 3.73	112.3 ± 1.6	
Pyruvic acid	74.49 ± 8.08	77.6 ± 4.56	
L-Alanine	280.62 ± 10.49	298 ± 10.8	300 ± 26
Lactic acid	2508.12 ± 27.36	2538 ± 2.9	
3-Hydroxybutyric acid	128.47 ± 6.04	138 ± 1.7	
Glycine	248.59 ± 6.96	244.6 ± 6.7	245 ± 16
L-Valine	159.72 ± 5.99	177.7 ± 7.7	182.2 ± 10.4
L-Leucine	97.77 ± 3.29	101.0 ± 5.6	100.4 ± 6.3
Isoleucine	49.60 ± 1.33	55.4 ± 2.1	55.5 ± 3.4
GABA	0.41 ± 0.06	0.345 ± 0.0572	
Succinic acid	2.33 ± 0.10	2.25 ± 0.01	
L-Proline	173.27 ± 4.48	169.1 ± 8.4	177 ± 9
Fumaric acid	0.84 ± 0.10	0.749 ± 0.076	
L-Methionine	15.04 ± 0.48	20.9 ± 1.6	22.3 ± 1.8
L-Serine	82.35 ± 2.16	91.2 ± 4.9	95.9 ± 4.3
L-Threonine	93.11 ± 2.78	118.3 ± 3.7	119.5 ± 6.1
L-Phenylalanine	48.26 ± 1.45	50.5 ± 2.3	51 ± 7
Hippuric acid	2.58 ± 0.18	2.31 ± 0.26	
L-Aspartic acid	4.43 ± 0.60	6.74 ± 2.3	
L-Glutamic acid	56.77 ± 2.32	59.2 ± 8.4	
Indole-propionic acid	0.44 ± 0.11	0.578 ± 0.033	
L-Lysine	128.47 ± 3.34	144.9 ± 6.4	140 ± 14
L-Histidine	51.85 ± 2.41	68.3 ± 2.6	72.6 ± 3.6
L-Tyrosine	48.42 ± 1.19	56.5 ± 1.8	57.3 ± 3.0

Altogether, we established a targeted metabolomics protocol for the quantification of 120 gut-bacteria-produced metabolites using a total of 52 isotopically labeled internal standard compounds. We report LOQ and linear quantification range for each of the metabolites to facilitate the analysis of biological samples.

### Quantification of gut microbiota-derived metabolites in the plasma and liver

**Fig. 3:**
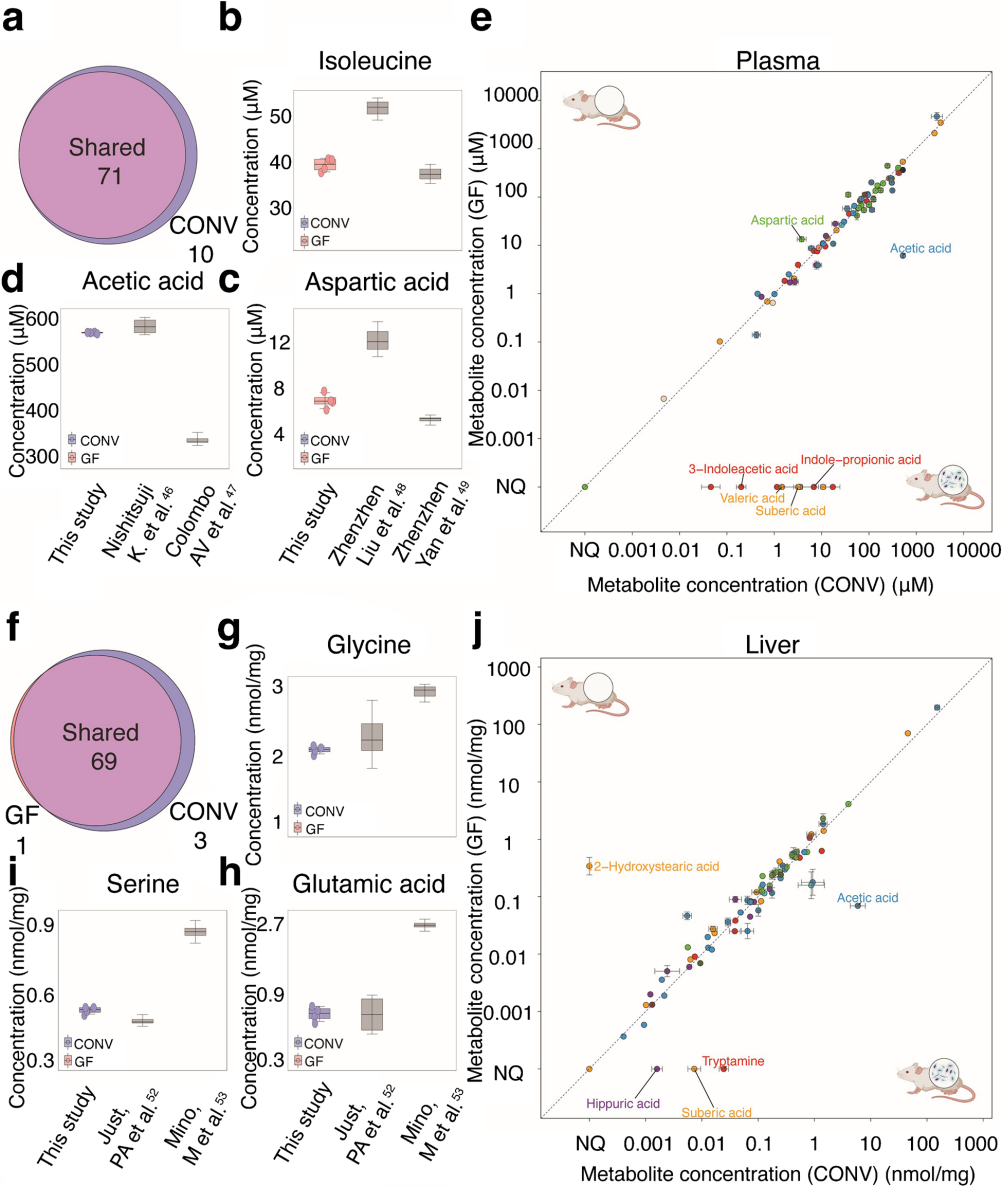
Metabolites quantified in plasma and liver tissues of CONV and GF animals. **a** Venn diagram representing the number of quantified metabolites in GF and CONV animals plasma samples. **b**–**d** Measured and previously reported concentration range of **b** isoleucine, **c** aspartic acid, and **d** acetic acid in plasma samples from CONV mice. **e** Scatter plot of metabolites quantified in plasma samples of conventional and GF animals. **f** Venn diagram representing the number of quantified metabolites in GF and CONV animals in liver samples. **g**–**i** Measured and reported concentration range of **g** glycine, **h** glutamic acid, and **i** serine in liver samples from CONV mice. **j** Scatter plot of metabolites quantified in liver samples of CONV and GF animals [46–51].

To demonstrate the utility of the developed targeted metabolomics method for quantifying microbiota-derived metabolites in vivo, we applied it to samples from six conventional (CONV) and six germfree (GF) C57BL/6 mice of mixed sex (Supplementary Table S4). Using the established GC-MS/MS protocol, we detected 86 metabolites in plasma samples in both groups of mice, of which we could quantify 81 (Fig. 3a). Measured concentrations align well with previously reported values, providing an important validation of the established method. For example, we measured a plasma concentration of 519.9 ± 8.2 µM for acetic acid in CONV mice, which aligns with previously reported concentrations of 101 µM and 580 µM (Fig. 3b) [44, 45]. Further, amino acid concentrations in the plasma of GF mice, such as isoleucine (mean, 34.2 ± 4.2 µM; reported range, 29 µM and 50 µM) and aspartic acid (mean, 6.7 ± 0.8 µM; reported range, 5 µM and 11 µM), were within the range of previously reported concentrations (Fig. 3c, d) [46, 47].

Among the 81 quantifiable plasma metabolites, we detected ten solely in the plasma of CONV mice (Fig. 3a, Supplementary Table S5). Furthermore, 40 metabolites showed significant differences (*p*_adj_ < 0.05) between GF and CONV animals, illustrated in the scatter plot (Fig. 3e). Among the metabolites with higher plasma concentration in CONV animals are indole-acetic acid and indole-propionic acid, which are solely produced by microbes but not the host and, hence, should indeed not be detected in germfree animals [20]. Furthermore, microbiota-produced acetic acids, 2-ketoisocaproic acid, and valeric acids are more abundant in the plasma of CONV mice. Altogether, these data demonstrate that our developed method is capable of detecting microbiota-dependent metabolites in plasma samples and that the determined concentrations of specific metabolites are in agreement with previous reports.

Next, we wanted to test our protocol for the measurement of metabolites extracted from solid tissues. To this aim, we extracted metabolites from liver samples from the same animals using the bead-beating protocol previously described [52]. We detected 78 metabolites, 73 of which we could quantify. Measured concentrations also align well with previously reported values. For example, the concentration of glycine (mean, 2.2 ± 0.1 nmol per mg; reported range, 0.6 and 2.7 nmol per mg), glutamic acid (mean, 0.7 ± 0.1 nmol per mg; reported range, 1.2 and 2.1 nmol per mg), and serine (mean, 0.4 nmol ± 0.05 per mg; reported range, 0.32 and 1.00 nmol per mg) in CONV mice (Fig. 3g–i) [50, 51]. Three of the detected metabolites (hippuric acid, suberic acid, and tryptamine) were quantified only in the liver of CONV mice, whereas 12-hydroxystearic acid was only quantifiable in GF mice, which highlights the microbiome dependency of these metabolites. (Fig. 3j, Supplementary Table S6).

### Quantification of gut microbiota-derived metabolites along the intestine

**Fig. 4:**
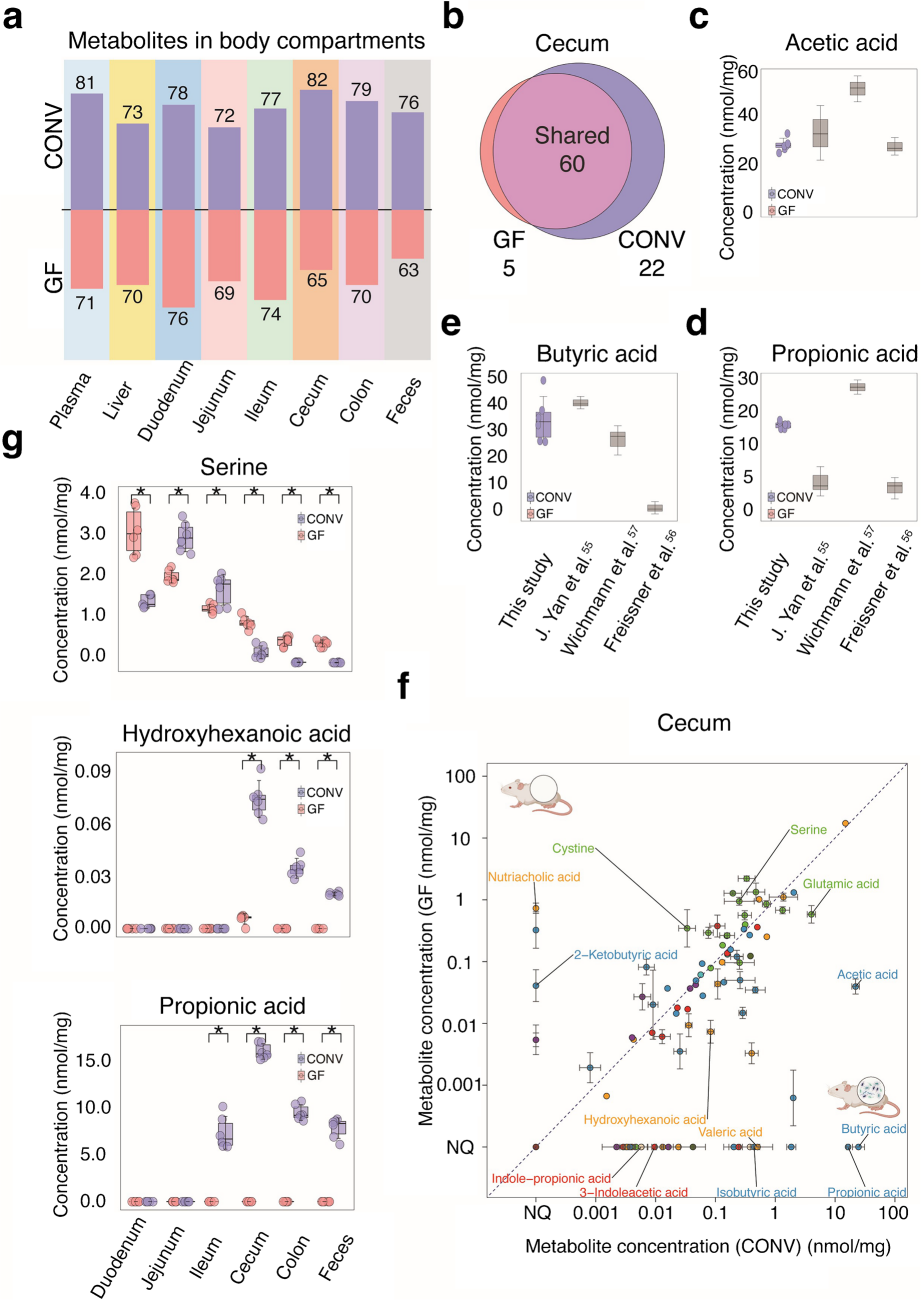
Metabolites quantified in intestinal content and tissues of CONV and GF animals. **a** Number of quantified metabolites across intestinal content and tissues of CONV and GF mice. **b** Venn diagram representing the number of quantified metabolites in GF and CONV animals in cecum samples. **c**–**e** Reported and measured concentration range of **c** acetic acid, **d** propionic acid, and **e** butyric acid in the cecum of GF and CONV animals. **f** Scatter plot of metabolites quantified in the cecum of CONV and GF animals. The concentration of all 82 quantified metabolites is represented as mean concentrations (nmol per mg) from six mice in each group. **g** Serine, hydroxyhexanoic acid, and propionic acid concentrations in the intestinal content along the gut of GF and CONV mice. An independent samples *t*-test was used to compare metabolite levels between the two groups, *p_adj_ < 0.05 [53–56].

Since we measured microbiota-dependent differences in the concentration of specific metabolites in plasma and liver, we next aimed to quantify microbiota-produced metabolites directly in the complex intestinal tract of GF and CONV mice. To this aim, we analyzed the intestinal content of different sections of the intestine (duodenum, jejunum, ileum, cecum, colon, and feces) to quantify metabolites at the site of gut microbial activity. In total, we analyzed 72 samples, including six mouse replicate samples per intestinal section for either group (i.e., CONV and GF). From our panel of 120 targeted metabolites, we could quantify between 63 and 82 in a given intestinal section and mouse group, with site-specific and colonization-dependent differences in detected metabolites (Fig. 4a, Supplementary Table S7-S12).

We first focused our analysis on the cecum because most microbial metabolic activity is expected in this intestinal section [53]. We detected a total of 97 cecal metabolites, 82 of which we could quantify, with 5 and 22 metabolites only quantifiable in the cecum of GF and CONV mice, respectively (Fig. 4b, Supplementary Table S8). For 80 of the 82 quantified metabolites, concentrations remained within the linear range of the method (maximum measured concentration = 987 µM). However, acetic acid and propionic acid levels exceeded this range in three cecal samples from conventional mice. Therefore, we re-measured these samples after doubling the volume of the derivatization solution during sample preparation to dilute the samples. Nevertheless, one acetic acid sample remained above the linear range post-dilution and was therefore excluded from further analysis. Measured concentrations also align well with previously reported values. For example, the concentration of acetic acid (mean, 22.1 ± 3.2 nmol per mg; reported range, 20 and 60 nmol per mg), butyric acid (mean, 24.6 ± 5.7 nmol per mg; reported range, 16 and 48 nmol per mg), and propionic acid (mean, 16,5 ± 0.9 nmol per mg; reported range, 4.00 and 20.00 nmol per mg) in CONV mice was within the previously reported range (Fig. 4c, d, and e) [54–56]. Among the 82 quantified metabolites, 57 were significantly different between CONV and GF animals (*p*_adj_ < 0.05), illustrated in the scatter plot (Fig. 4f, Supplementary Table S10) with key microbiota-dependent metabolites labeled, such as acetic acid, valeric acid, propionic acid, indole-propionic acid, isocaproic acid, and isovaleric acid. The elevated levels of these microbiota-associated metabolites in CONV mice, alongside higher amino acid concentrations in GF mice (e.g., proline concentration, GF = 2.2 ± 0.2 nmol/mg vs. CONV = 0.33 ± 0.10 nmol/mg), can be explained by microbial metabolism. Notably, we detected several microbiota-produced SCFA in the cecum, such as butyric acid and propionic acids, that were absent in the plasma of CONV mice (Fig. 3c). This difference is likely due to their rapid consumption by enterocytes in the gut epithelium and the liver.

To further illustrate gut bacterial metabolism, we performed measurements along the intestinal tract (Fig.  4 g, Supplementary Figs. 1, 2, 3, 4 and 5). For example, to illustrate the lack of microbial consumption or transformation of amino acids in the distal colon of GF mice, we show serine concentrations along the gut (Fig.  4 g). Serine concentrations are significantly higher (*p*_adj_ < 0.05) in the distal colon of GF compared to CONV mice, reflecting the absence of a microbiota that consumes serine in the gut GF mice. Contrarily, to demonstrate microbial metabolite production, we show that hydroxyhexanoic and propionic acid concentrations have an increasing gradient along the intestinal tract—particularly enriched in the cecum and colon of CONV mice (Fig.  4 g). These patterns reflect known microbial colonization zones and emphasize region-specific bacterial fermentation.

### Determine method precision and accuracy

**Fig. 5:**
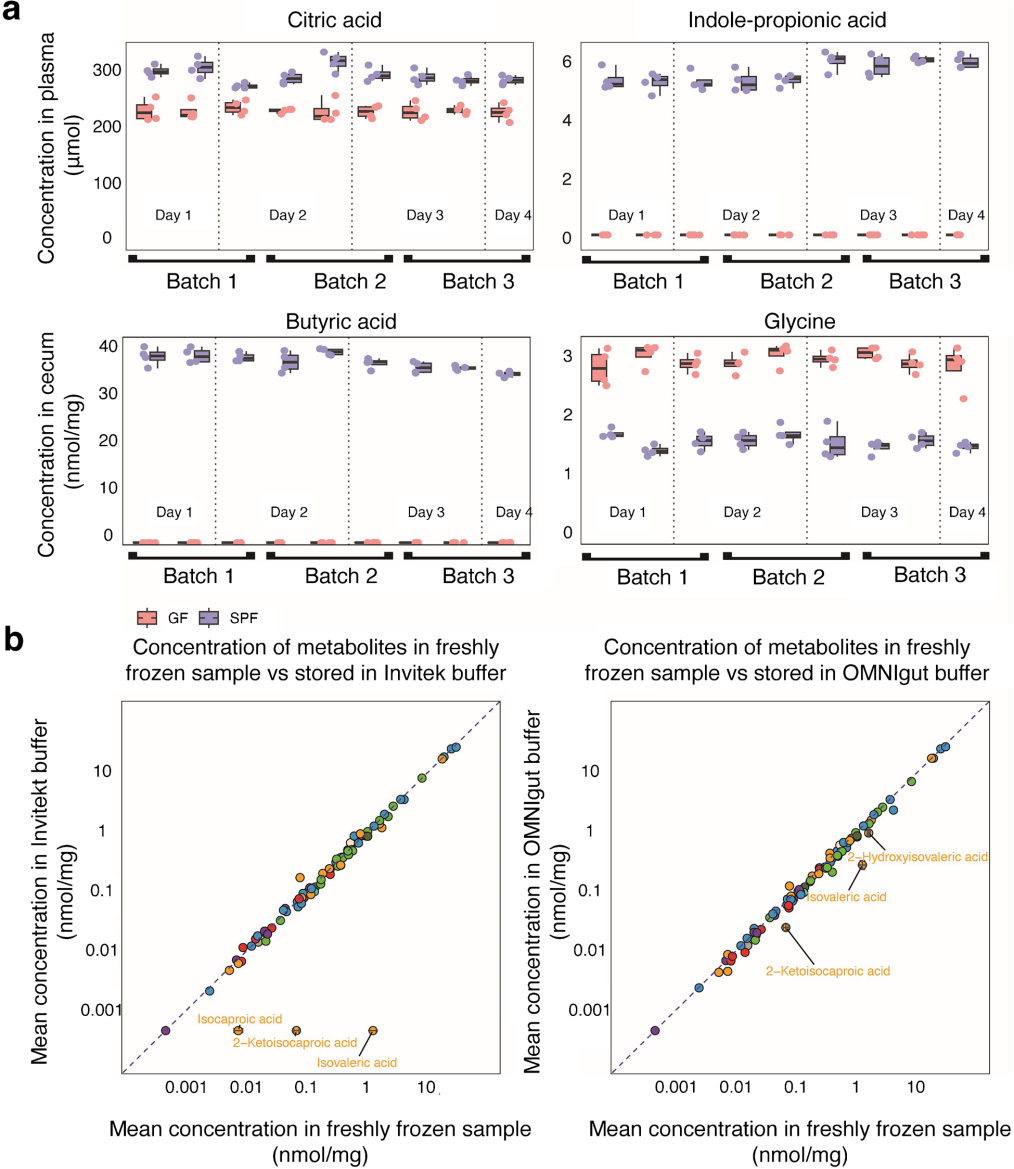
Metabolites quantified in cecum and plasma of CONV and GF mice analyzed in different measurement batches to assess intra- and interday measurement precision and metabolites quantified in cecal samples of conventional animals stored under three different preanalytical conditions. **a** Box plots of citric acid and indole-propionic acid quantified in the plasma of CONV and GF animals, and butyric acid and glycine quantified in the cecum of CONV and GF animals. The same samples were processed in three independent batches (each containing four identical replicates) on three different days, and each processed batch was measured by GC-MS/MS three times (two times on the same day and once on the subsequent day). **b** Scatter plot of quantified metabolites in cecum sample stored in Invitek and OMNIgut stabilization buffers and in a freshly frozen sample. The abundances of all 82 quantified metabolites are represented as mean concentrations from four replicates in each group.

To assess the intraday and interday precision of the developed method, we extracted three batches of four replicates of the same sample (i.e., plasma and cecum from both germfree and conventional mice) on three different days. We then measured each sample batch twice on the same day to assess intraday measurement precision and once on the subsequent day to assess interday measurement precision. To illustrate method precision, representative metabolites covering a broad concentration range in plasma and intestinal content sample matrices are displayed (Fig. 5a). For plasma, indole-propionic acid, solely produced by the gut microbiota, and citric acid, produced by microbes and the host, were selected to illustrate different physiological concentrations. For the cecum, butyric acid and glycine are shown. Precision, quantified as %CV, was determined for citric acid: CONV: interday CV = 6.14, intraday CV = 4.77, and GF: interday CV = 5.50%, intraday CV = 5.50%; for indole-propionic acid: CONV interday CV = 6.40%, intraday CV = 7.90%, and for GF: not quantified; for butyric acid: CONV interday CV = 5.65%, intraday CV = 4.27%, and for GF: not quantified; and for glycine: CONV: interday CV = 11.09, intraday CV = 13.14, and for GF: interday CV = 9.64, intraday CV = 7.03. All these values fall within the acceptable range (CV < 20%) defined by current analytical guidelines (Supplementary Table S15) [57]. In total, we could assess the precision for 80 distinct metabolites in all four sample matrices (i.e., plasma and cecum from both germfree and conventional mice), and for all of them, we determined an intraday and interday CV < 20% (Supplementary Table S15).

### Quantification of gut microbiota-derived metabolites in DNA/RNA stabilization buffers

Next, we tested the capacity of the developed method to detect gut-bacterial metabolites in intestinal content stored in DNA stabilization buffers. To this aim, we pooled and homogenized cecal material from CONV mice and equally distributed four aliquots in three different storage conditions: (i) freshly frozen samples without any additional solution, (ii) samples stored in Invitek stabilization buffer, and (iii) samples stored in OMNIgene gut stabilization buffer. We then froze the samples at −70 °C and analyzed them using our developed GC-MS/MS protocol. We detected a total of 96 compounds in freshly frozen samples, among which we could quantify 82.

For samples stored in Invitek buffer, we could quantify 79 of the 82 metabolites quantified in fresh-frozen samples, with isocaproic acid, 2-ketoisocaproic acid, and isovaleric acid not being detected (Fig. 5a, Supplementary Table S13). All other metabolites showed comparable concentrations between the two sample storage conditions (e.g., butyric acid in freshly frozen samples = 23.9 ± 0.41 nmol/mg compared to 21.35 ± 1.35 nmol/mg in Invitek buffer and alanine in freshly frozen samples = 1.63 ± 0.09 nmol/mg compared to 1.65 ± 0.1 nmol/mg in Invitek buffer).

In contrast, we could quantify all the 82 metabolites in OMNIgut that we also quantified in freshly frozen samples. However, we also found three metabolites that had significantly (*p*_adj_ ≤ 0.5) lower concentration in OMNIgut stabilization buffer compared to fresh-frozen samples, including 2-ketoisocaproic acid, 2-hydroxyisovaleric acid, and isovaleric acid (Fig. 5b, Supplementary Table S13).

Furthermore, we noticed that measured metabolite intensities in Invitek stabilization buffer are generally lower than those from freshly frozen and OMNIgut buffers. The extensive use of internal standard compounds compensates for this for the quantification of metabolites, but the limit of detection is reduced.

## Conclusions

We have developed and validated a GC-MS/MS method for the simultaneous quantification of 120 chemically diverse gut microbiota-derived metabolites across multiple biological matrices. By employing multiple-reaction-monitoring (MRM) and incorporating 52 isotopically labeled internal standards, our approach ensures high specificity, sensitivity, and quantitative accuracy for a broad range of metabolite classes, including SCFAs, BCFAs, organic acids, amino acids and their derivatives, indole compounds, and lipid-like molecules.

To demonstrate the versatility and biological relevance of our method, we applied it to samples from germfree and conventionally raised mice. The method successfully captured microbiota-dependent metabolic signatures across systemic and intestinal compartments, with key metabolite concentrations closely aligning with established literature values. Our results also revealed site-specific differences in microbial metabolism along the gastrointestinal tract, reflecting known microbial colonization zones and metabolic microbiota-host interactions. Furthermore, we evaluated the compatibility of the method with DNA/RNA stabilization buffers commonly used in microbiome studies. We demonstrated that the majority of metabolites can be accurately quantified in two commonly used DNA/RNA stabilization solutions, OMNIgut and Invitek buffer.

We acknowledge that variability in water content between samples can introduce imprecision, particularly for metabolites whose concentrations are sensitive to tissue hydration, such as highly polar small molecules and SCFAs. In this study, metabolite concentrations were normalized to wet tissue weight, which is a common and practical approach in targeted metabolomics. However, this normalization does not account for inter-sample differences in water content, which may lead to slight over- or underestimation of metabolite concentrations in samples that are more or less hydrated than average. Alternative normalization strategies, such as dry-weight normalization or incorporation of tissue water content measurements, could further reduce this source of variability, but were not applied in the current study. We therefore recommend interpreting the provided concentrations with this limitation in mind.

Altogether, the developed GC-MS/MS method offers an analytical protocol for microbiome research, facilitating accurate quantification of gut-derived metabolites. It provides a metabolomic tool for future studies aiming at the quantification of microbiota-host metabolic interactions and the effects of dietary or microbial interventions on host physiology.

## Supplementary Information

Below is the link to the electronic supplementary material.

Supplementary Material 1 (JPG 425 KB)

Supplementary Material 2 (JPG 351 KB)

Supplementary Material 3 (JPG 375 KB)

Supplementary Material 4 (JPG 451 KB)

Supplementary Material 5 (JPG 395 KB)

Supplementary Material 6 (XLSX 198 KB)

## Data Availability

All processed data is available in the supplementary tables as indicated throughout the manuscript, and raw data have been deposited on Metabolights (accession number MTBLS12911). All code is available on GitLab (https://github.com/ZimmermannLab/gcms-gut-metab-quant).
