# Supplementary figures and images for: Development of a GC-MS/MS method to quantify 120 gut microbiota-derived metabolites
Source: Anal Bioanal Chem. 2025 Dec 15;418(4):1035–54. doi: 10.1007/s00216-025-06256-6 (PMC12901100; doi:10.1007/s00216-025-06256-6)

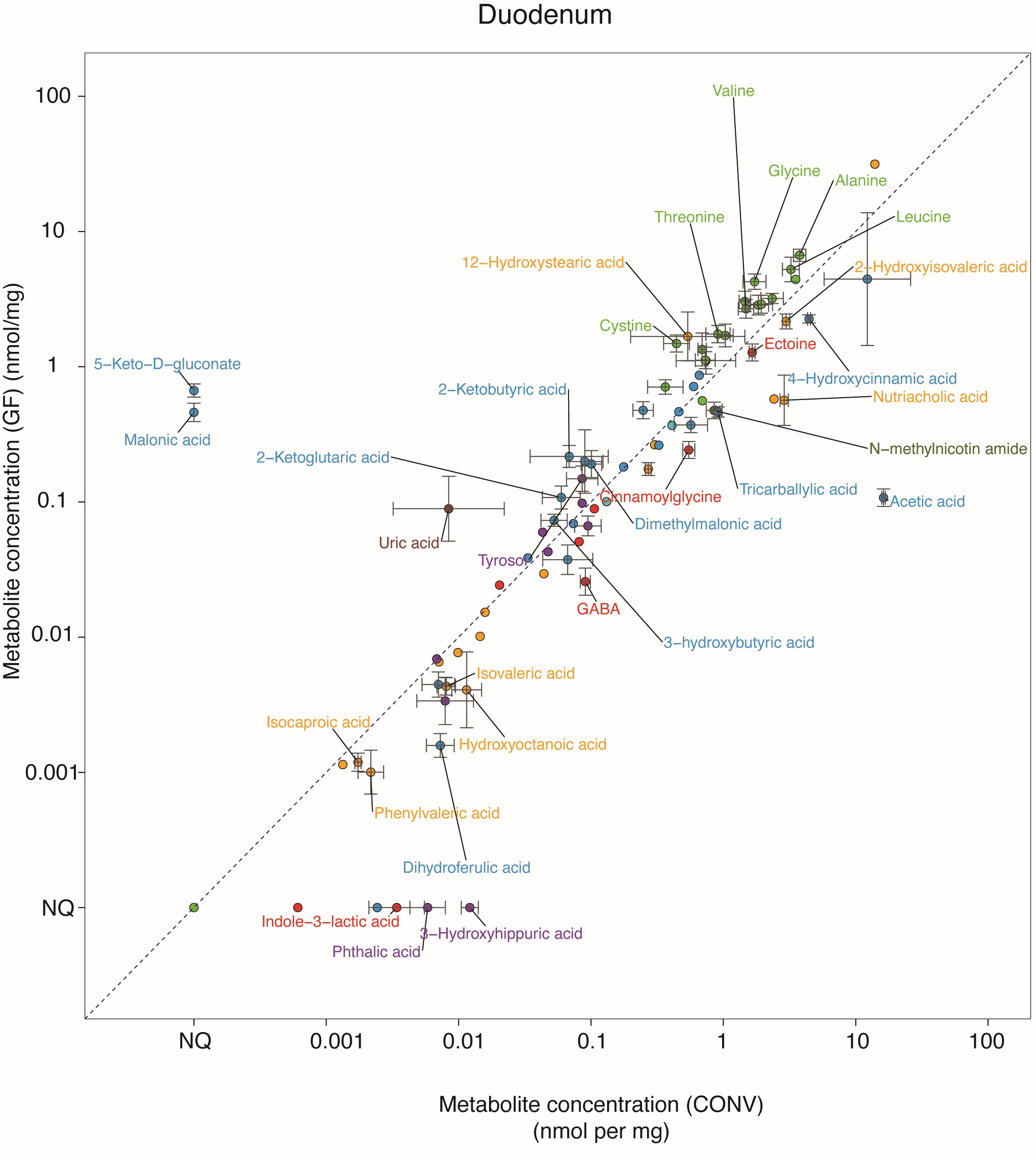

Supplement: Supplementary file 1 — Supplementary Material 1 (JPG 425 KB) [file 216_2025_6256_MOESM1_ESM.jpg]

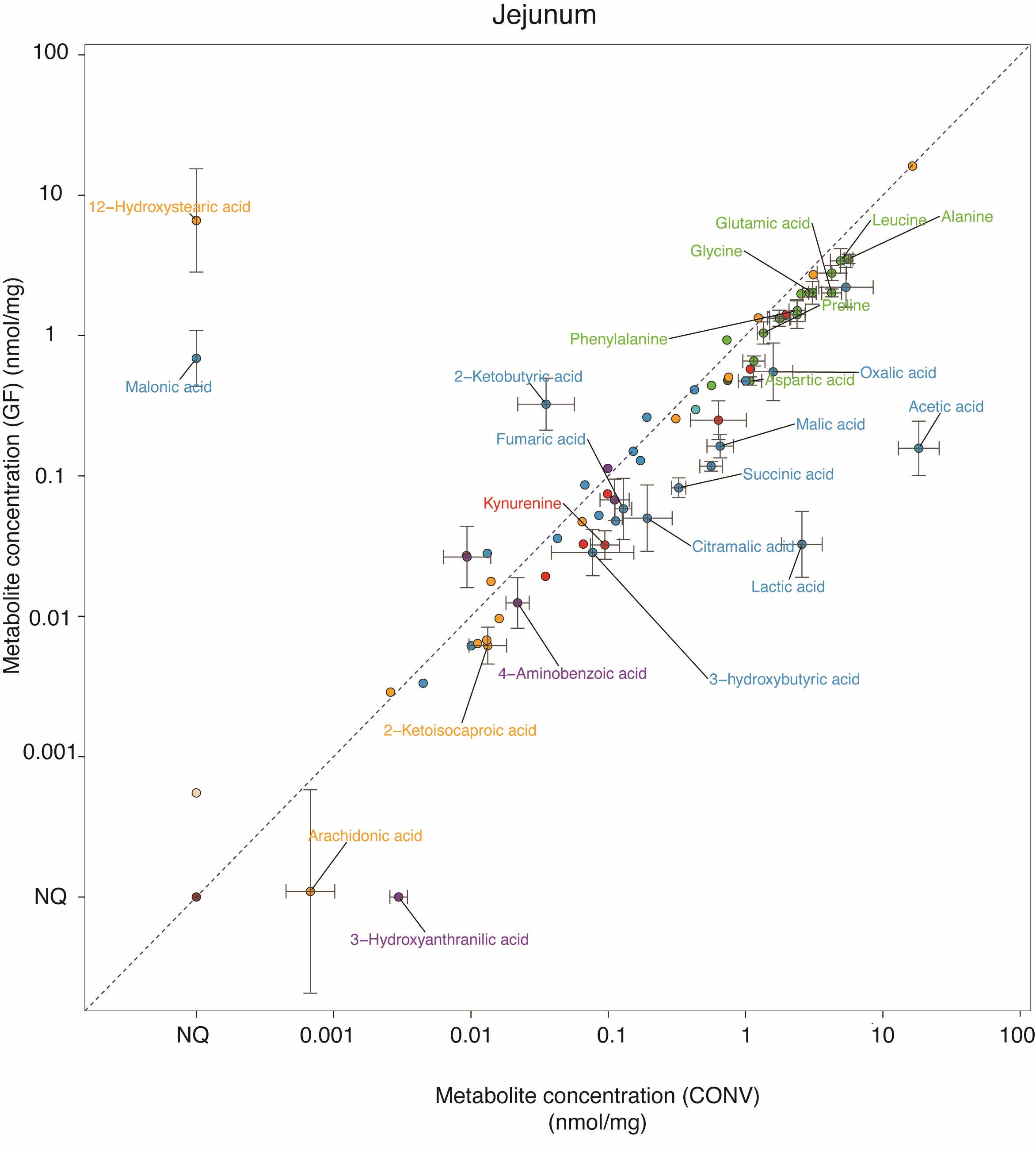

Supplement: Supplementary file 2 — Supplementary Material 2 (JPG 351 KB) [file 216_2025_6256_MOESM2_ESM.jpg]

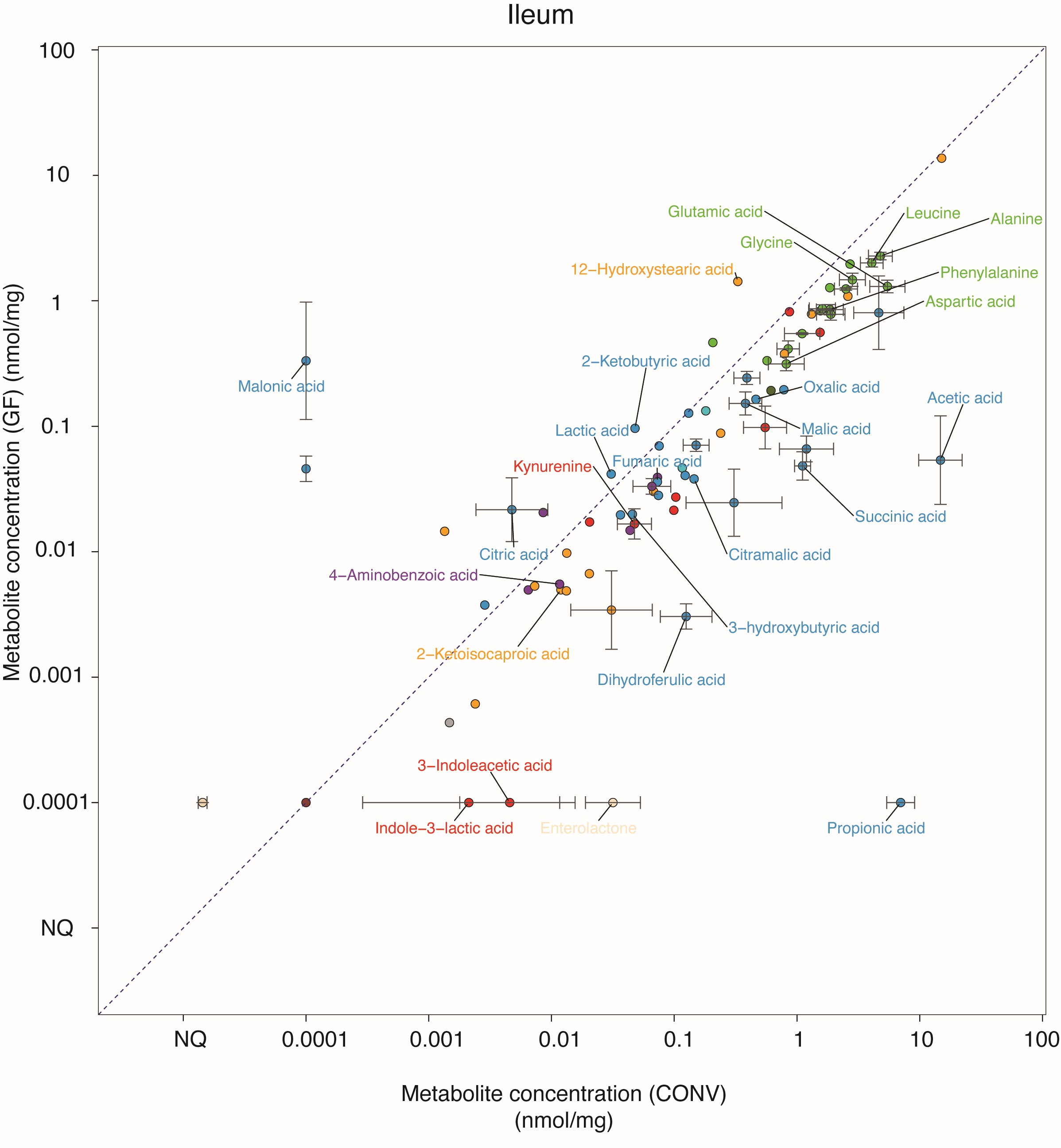

Supplement: Supplementary file 3 — Supplementary Material 3 (JPG 375 KB) [file 216_2025_6256_MOESM3_ESM.jpg]

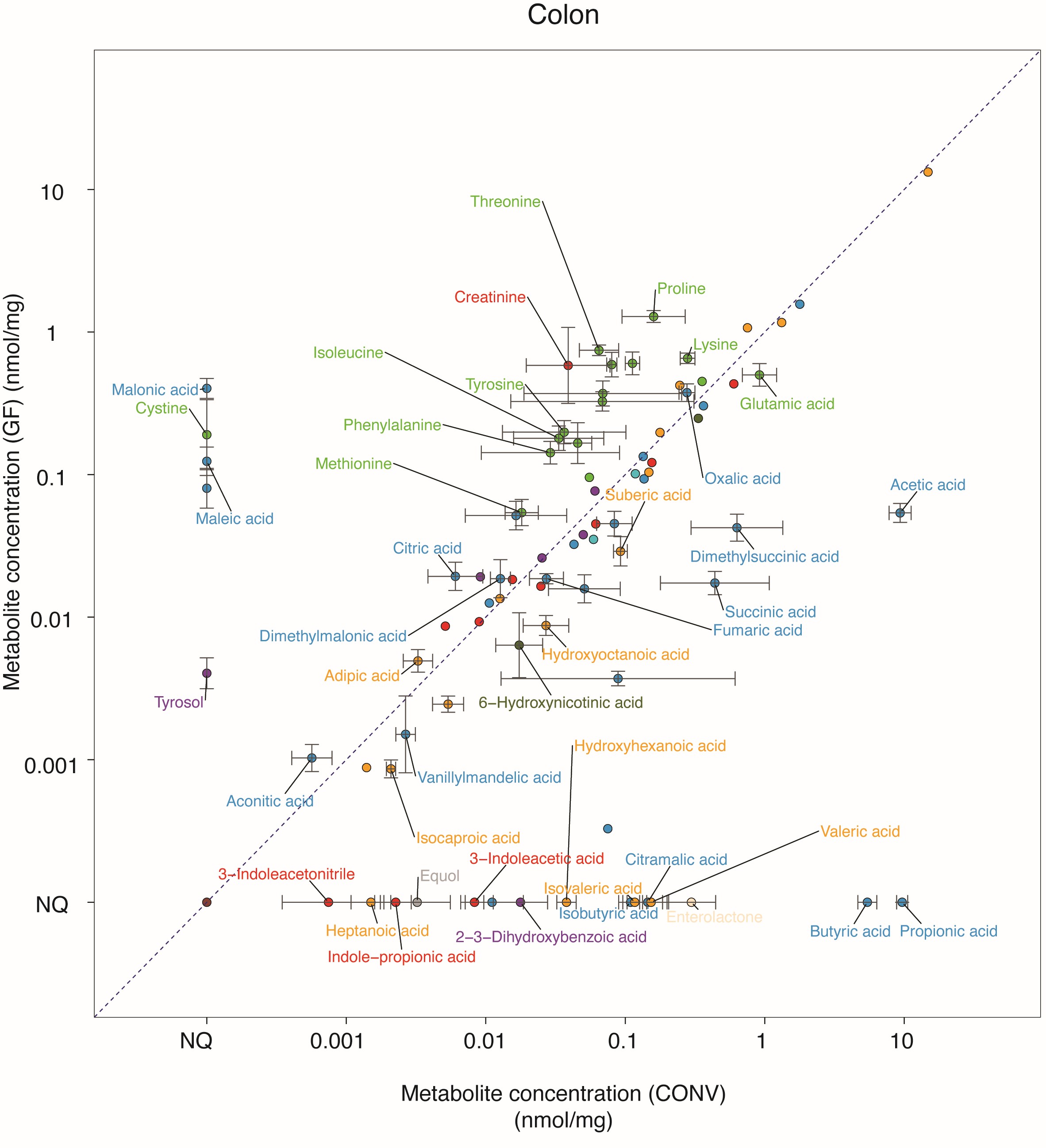

Supplement: Supplementary file 4 — Supplementary Material 4 (JPG 451 KB) [file 216_2025_6256_MOESM4_ESM.jpg]

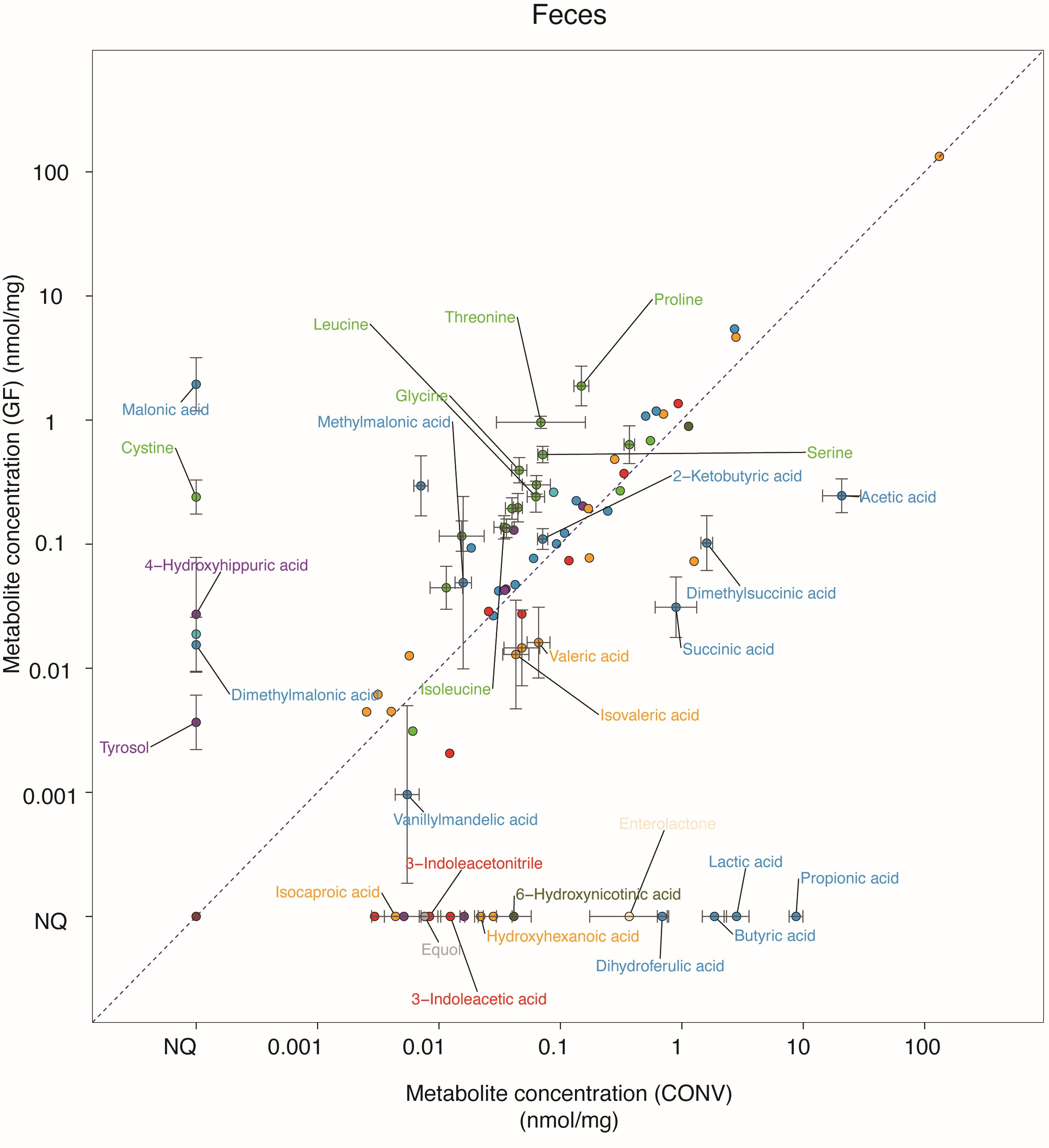

Supplement: Supplementary file 5 — Supplementary Material 5 (JPG 395 KB) [file 216_2025_6256_MOESM5_ESM.jpg]
